# Supplementary material for: Correlations between human mobility and social interaction reveal general activity patterns
Source: PLoS One. 2017 Dec 13;12(12):e0188973. doi: 10.1371/journal.pone.0188973 (PMC5728524; doi:10.1371/journal.pone.0188973)
Supplement: S1 Text — (PDF) [file pone.0188973.s001.pdf]

This is a short note, which describes the format of the attached data. The data is saved in json format and includes the activity series of all the users in the four channels. The keys in the json file is the anonymized user names and the value associated with each key is the time series. The time series is represented as a list. The elements of the lists corresponds to the activity in the four channels in the order: call, SMS, mobility, and social proximity. The values 1 and 0 correspond to respectively activity and inactivity. Time bins that are not trusted (as explained in the supplement) is labelled with 1. The offset of the time series is at midnight the 26/09/2013 (Danish time), which is prior to most subjects obtaining their phones. Therefore, the beginning of most time series will be labelled by 1's.
